# PRES in the course of hemato-oncological treatment in children

**DOI:** 10.1007/s00381-017-3664-y

**Published:** 2017-12-02

**Authors:** Katarzyna Musioł, Sylwia Waz, Michał Boroń, Magdalena Kwiatek, Magdalena Machnikowska-Sokołowska, Katarzyna Gruszczyńska, Grażyna Sobol-Milejska

**Affiliations:** 1 Department of Pediatric Oncology, Hematology and Chemotherapy, Medical University of Silesia, Upper Silesia Children’s Care Health Centre, 16 Medykow Str, 40-752 Katowice, Poland; 2 Department of Diagnostic Imaging, Medical University of Silesia, Upper Silesia Children’s Care Health Centre, Katowice, Poland

**Keywords:** Paediatric oncology, Neurotoxicity of therapy, Chemotherapy neurotoxicities

## Abstract

**Introduction:**

Posterior reversible leukoencephalopathy syndrome (PRES) is a clinical syndrome of varying aetiologies, characterised by acute neurological symptoms of brain dysfunction with MRI abnormalities in posterior cerebral white and grey matter. In most cases, symptoms resolve without neurological consequences.

**Aim:**

The aim of this paper is the analysis of predisposing factors, clinical outcomes and radiological features of PRES in eight children with hemato-oncological disease.

**Material and methods:**

We analysed the medical records of eight hemato-oncological patients aged from 3.0 to 16.1 years. The mean of age at primary diagnosis was 8.5 years.

**Results:**

All patients had both clinical and radiological PRES features. Seven out of eight underwent intensive chemotherapy regimens. Time elapsed from start of treatment to the occurrence of PRES ranged from 6 to 556 days. In one case, PRES occurred before chemotherapy and was the first symptom of cancer. Most (six of eight) patients had history of hypertension (> 95pc) and some (two of eight) occurred electrolyte imbalance—mainly hypomagnesaemia. Patients presented headache (seven of eight), disturbances of consciousness (six of eight), seizures (six of eight), visual changes (four of eight) and vomiting (three of eight). MRI demonstrated abnormalities in seven children: typical cerebral oedema in the white matter of the occipital to the parietal lobes. Most patient lesions in the MRI shrunk after 4 weeks, and clinical symptoms of PRES disappeared completely within a few hours to few days.

**Conclusion:**

PRES may complicate oncological treatment in children. Hypertension is the most important risk factor of PRES. PRES should be included in differential diagnosis in all patients with acute neurological symptoms.

## Introduction

Posterior reversible leukoencephalopathy syndrome (PRES) is a clinical syndrome of varying aetiologies with similar neuroimaging findings and clinical symptoms. It is characterised by acute neurological symptoms such headache, visual changes, vomiting, seizures and disturbances of consciousness associated with magnetic resonance imaging abnormalities in posterior cerebral white and grey matter [1, 2]. The cerebral abnormalities are primarily located within the parietal and occipital lobes. Magnetic resonance imaging (MRI) is a gold standard in PRES [3].

The posterior reversible leukoencephalopathy syndrome was first described as “reversible posterior leukoencephalopathy syndrome” in 1996 by Hinchey et al. [4]. In 2000, Sean O. Casey proposed the current name of posterior reversible encephalopathy syndrome; however, the terminology debate is still ongoing.

Patients with PRES often require intensive care, although the prognosis is usually favourable and the syndrome resolves without permanent neurological damage [3]. However, unlike what the name implies, irreversible PRES-induced MRI or clinical damage was reported. Similarly, there are reports of other locations of cerebral lesions in PRES patients (the frontal or temporal lobes, brainstem, basal nuclei) [2, 5, 6].

The underlying mechanism of PRES is still under investigation. Two theories have been put forth: the vasogenic theory and cytotoxic theory. Whereas the former proposes that an acute increase in blood pressure overrides the autoregulation of cerebral mean arterial pressure, leading to vasodilatation and a non-cell autonomous endothelial insult. The latter postulates that a direct toxic effect on the endothelial cells of the cerebral vasculature results in cell autonomous endothelial dysfunction. In both cases, the end result is the diffusion of plasma proteins and cells into the extracellular space and subsequent cerebral oedema [7].

A number of predisposing factors and conditions have been implicated, including cytostatic and immunosuppressive therapies, toxaemia and solid organ and bone marrow transplantation, as well as kidney disease [2, 8, 9]. According to the research, hypertension is the main risk factor of PRES [10].

## Aim

The aim of this paper is to analyse predisposing factors, clinical outcomes and radiological features of PRES diagnosed in children with hemato-oncological diseases.

## Material and methods

We reviewed clinical records of patients treated at our paediatric hemato-oncological centre in 2008–2016. A retrospective analysis indicated eight patients (three girls, five boys) with PRES based on radiological findings and clinical assessment. Mean age at presentation was 8.8 years (range from 3.0 to 16.1 years). Primary diagnosis in children were ALL pre-B (acute lymphoblastic leukaemia) (two of eight), granulocytic sarcoma transformed to AML (acute myeloblastic leukaemia) (M2) CNS+ (one of eight), hepatoblastoma (one of eight), nephroblastoma (one of eight), neuroblastoma III (one of eight), primitive neuroectodermal tumour (PNET) (one of eight) and aplastic anaemia (one of eight). The mean of age at primary diagnosis was 8.5 years.

The collected data included patient demography, predisposing conditions, clinical symptoms, neurology assessment findings, neuroimaging scans and laboratory test results, such as serum electrolytes, cerebrospinal fluid (CSF) analysis, as well as blood pressure measurements at the onset of PRES. Arterial hypertension was defined as systolic or diastolic blood pressure values exceeding the 95th percentile for the appropriate age group.

MRI was performed in seven patients, CT in four cases. All MRI studies included axial T1- and T2-weighted images, axial fluid-attenuated inversion recovery (FLAIR) images and diffusion-weighted imaging sequences (DWI) including ADC (apparent diffusion coefficient) map. All scans were evaluated by two independent radiologists.

## Results

### Clinical manifestation

Clinical manifestation varied significantly among the studied patients. The most common symptom was headache, reported by seven of eight patients. Six patients developed seizures, including three cases of long-term *status epilepticus*. Fifty per cent (four of eight) patients presented with visual disturbances. Quantitative consciousness disorders (drowsiness, lethargy) were noticed in six patients, whereas three patients presented with qualitative consciousness disorders (visual and/or auditory hallucinations). Vomiting occurred in three patients. Four patients required intensive care, the ICU hospitalisation ranged from 1 to 7 days; the mean duration of ICU hospitalisation was 4.25 days. Clinical symptoms of PRES resolved completely within hours to days. In six patients, PRES occurred when receiving chemotherapy. Treatment duration until the onset of PRES was relatively long in five patients ranging from 55 to 150 days (mean of 84.4 days). There was a single case of PRES onset 556 days following cancer treatment commencement, a single case (female with aplastic anaemia) of PRES onset 6 days following myeloablative treatment commencement and a single case of PRES onset prior to chemotherapy as a first sign of malignancy (PNET) (Table 1).

### Radiological features

**Fig. 1:**
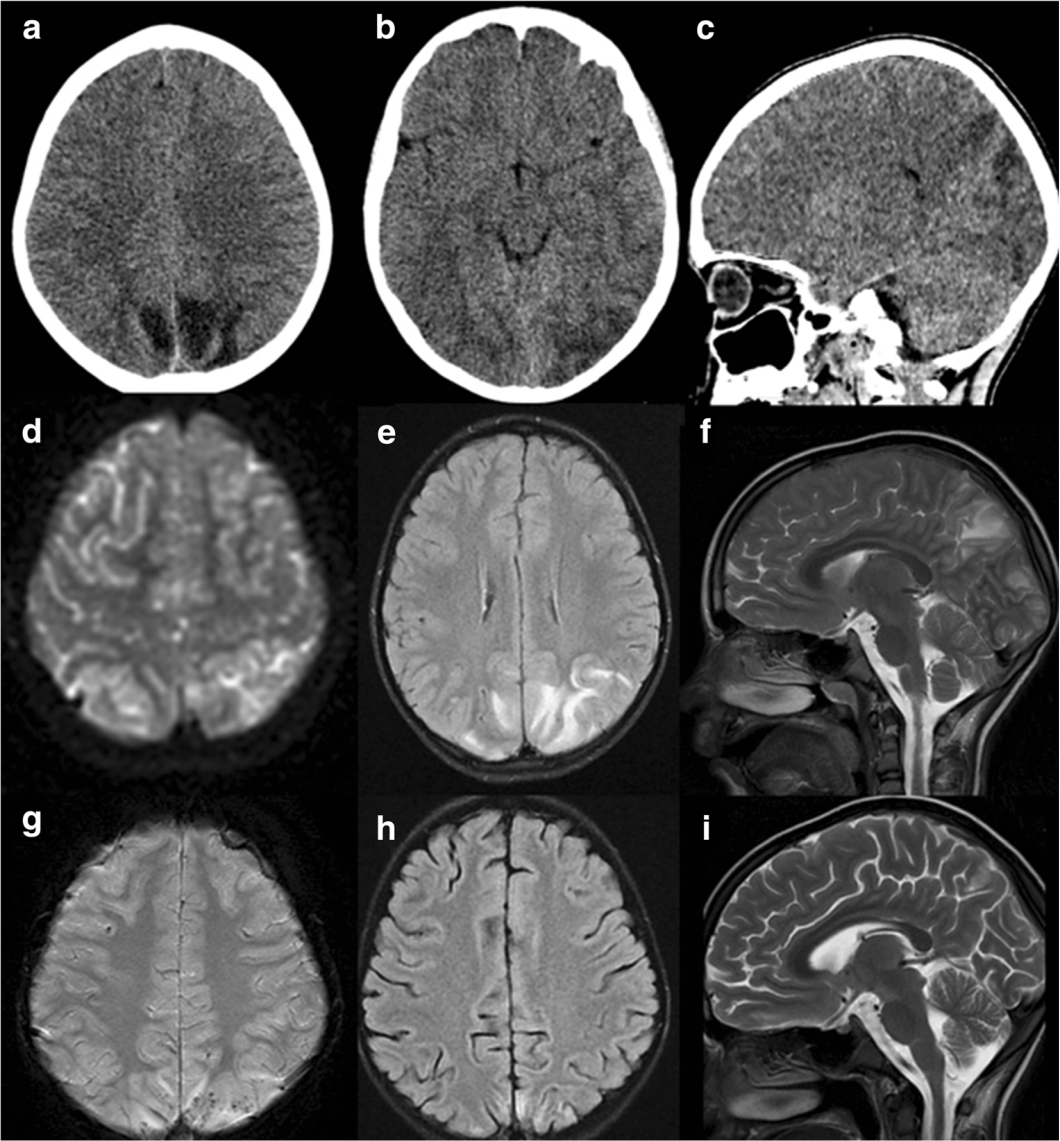
**a–c** Initial head CT axial plane (**a** and **b**) and sagittal multiplanar reconstruction (**c**): hypodense cortical-subcortical areas in the parietal lobes bilaterally and in the left occipital lobe—ischaemic changes with oedema. **d**–**f** Head magnetic resonance (MRI) in same day, axial diffusion-weighted image (DWI) (**d**): restriction of water diffusion, axial fluid-attenuated inversion recovery (FLAIR) image (**e**) and sagittal T2/weighted image (**f**): hyperintense signal in the parietal and occipital lobes—regions of cortical/subcortical oedema, correlating with PRES. **g–i** Control head MRI after 3 months, axial susceptibility weighted image (SWI) (**g**): small haemorrhagic regions, axial FLAIR image (**h**): substantial regression of changes in occipital lobes, sagittal T2/weighted image (**i**): small residual changes.

Magnetic resonance imaging was conducted in seven of eight cases. MRI was carried out immediately following the first signs of PRES (range 0 to 5 days, mean time to MRI was 2.14 days). All scans were independently reviewed by two different radiology and diagnostic imaging consultants. The most common findings included lesions within the occipital lobes (all scanned patients), parietal lobes (all scanned patients), frontal lobes (six patients), temporal lobes (two patients) and cerebellum (two patients). There was a single case of brainstem involvement. Typical lesions of high-signal intensity on T2-weighted images and FLAIR were present in all patients. Grey matter involvement was confirmed in five cases. Focal enhancement after contrast infusion was present in two of seven patients. The follow-up MRI was carried out in six of seven previously scanned patients. Complete resolution of lesions previously seen in MRI was confirmed in five cases and there was one case of partial regression. CT (computer tomography) scan, performed initially in four of seven patients, was diagnostic only in one case of PRES (Fig. 1).

### Risk factors of PRES (Table 2)

#### Blood pressure

**Fig. 2:**
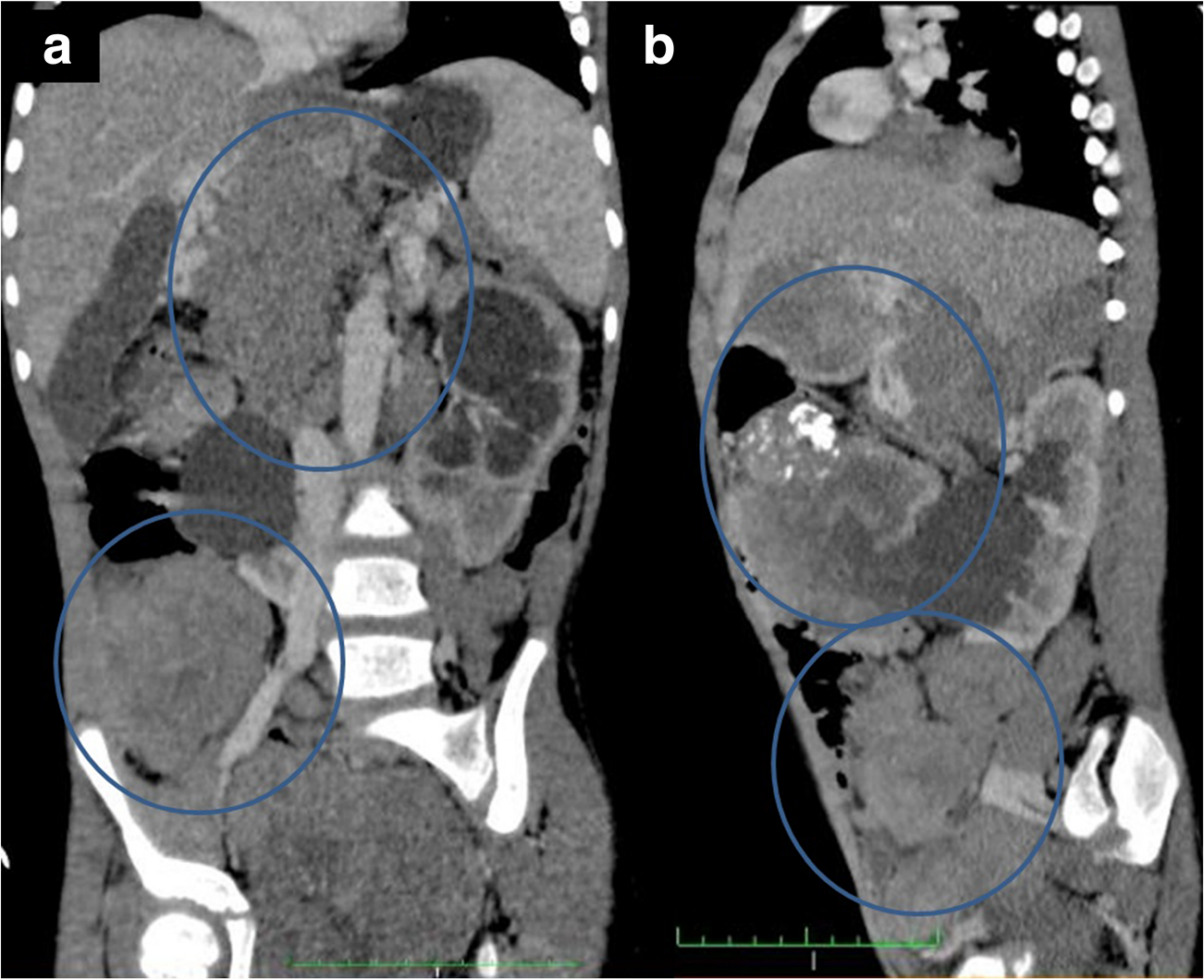
Abdominal and pelvic CT: coronal (**a**) and sagittal multiplanar reconstruction (**b**)—pathologic soft tissue mass in abdomen and pelvis (multifocal PNET) with calcifications, left kidney hydronephrosis

A sudden elevation of blood pressure (both systolic and diastolic) to values exceeding the 95th percentile (for their sex, age and height) was observed in six patients. One patient presented with prehypertension (90–95 percentile), whilst another one had normal blood pressure (50th–90thpercentile) at the onset of PRES. The mean peak systolic blood pressure was 150 mmHg (range 110–180 mmHg), whilst the mean peak diastolic pressure was 94 mmHg (range 70–110 mmHg). Only one patient (with primary diagnosis of nephroblastoma) had a history of hypertension and was on hypotensive treatment. Three patients developed hypertension secondarily to kidney disease, as a result of a tumour mass compressing renal blood vessels or urinary tract, which caused urinary retention within the pyelocalyceal system thus elevating blood pressure (Fig. 2). Corticosteroids were used in four cases. The female with aplastic anaemia most likely developed drug-induced hypertension, with cyclosporin and steroids.

**Table 1:** Demographic data and clinical features

Patient	Sex	Primary diagnosis	Age at primary diagnosis (years)	Period from treatment onset to PRES occurrence (days)	Symptoms at presentation of PRES					
					HT	H	S	AMS	VD	V
1	F	AML	8.5	150	+	+	+	+	+	
2	F	Hepatoblastoma	13.7	66	+	+	+	+		+
3	M	Nephroblastoma	3.3	556	+	+	+	+	+	
4	M	ALL	11.75	55		+	+	+		
5	M	Neuroblastoma	3.3	84	+	+			+	
6	M	PNET	8.5	0	+	+	+	+		+
7	M	ALL	2.8	67	+					+
8	F	Aplastic anaemia	16	6	+	+	+	+	+	

*F* female, *M* male, *AML* acute myeloblastic leukaemia, *ALL* acute lymphoblastic leukaemia, *PNET* primitive neuroectodermal tumour, *HT* hypertension, *H* headache, *S* seizures, *AMS* altered mental status, *VD* visual disturbance, *V* vomiting, + symptom present

**Table 2:** Risk factors for PRES

Patient	HT before PRES	Risk factors for high blood pressure	Other risk factors for PRES	Chemotherapy	
				Intravenous therapy	Intrathecal administration
1	1		Sepsis blood transfusion	ARA-C	ARA-C
2	0	Cisplatin Hypomagnesaemia	Sepsis blood transfusion	CDDP	
3	0	Kidney dysfunction Corticosteroids	Auto-HSCT blood transfusion	VCR, CBDCA	
4	0	Corticosteroids	Blood transfusion	VCR, ARA-C, L-ASP	MTX
5	0	Kidney dysfunction Cisplatin	Blood transfusion	CBDCA, VCR	
6	0	Kidney dysfunction			
7	0	Corticosteroids	Sepsis	VCR, L-ASP Daunorubicine	MTX
8	0	Corticosteroids, hypomagnesaemia CsA	Blood transfusion	ATG, CsA	

*HT* hypertension, *ARA-C* cytarabine, *CDDP* cisplatin, *VCR* vincristine, *CBDCA* carboplatin, *L-ASP* L-asparaginase, *MTX* methotrexate, *HSCT* haematopoietic stem cell transplantation, *ATG*,anti-thymocyte globulin, *CsA* cyclosporin

#### Laboratory findings

Serum electrolytes were tested in all our patients at the onset of PRES symptoms. The patients presented with serum magnesium ion levels falling in the middle of the range (0.69 mmol/L; min 0.38–max 0.85 mmol/L). Low serum magnesium level has been reported as a cause of hypertension in two PRES patients. Normal levels of sodium, potassium and calcium ions were reported.

### Diagnosis and treatment

The course of diagnosis of our patients with PRES symptoms has been considerably shortened in recent years (from a week to a few hours). Our results confirm that MRI is the most important and key element for the diagnosis of PRES (Table 3).

**Table 3:** The course of diagnosis of our patients with PRES symptoms

Patient	Year of diagnosis of PRES	Period from first signs to diagnosis	Treatment after symptoms of PRES occurred						Tests				Medical consultations
			Antifungal, antibacterial, antiviral	Antioedematous	Antiseizure	Antihypertensive	Neuroleptic	i.v. magnesium sulphate	EEG	CSF	CT	MRI	
4	2008	7 days	+	+	+	+			+	+	+	+	Neurological, cardiological Anaesthetic
2	2009	6 days	+	+	+	+		+	+		+	+	Neurological Cardiological Ophthalmologic
1	2011	6 days	+	+	+	+	+		+	+		+	NeurologicalPsychiatric
8	2013	3 days	+	+	+	+						+	Neurological Anaesthetic
3	2015	2 days		+	+	+			+		+		Neurological Anaesthetic
5	2015	The same day		+		+						+	Nephrological
6	2016	The same day		+	+	+					+	+	
7	2016	The same day		+		+						+	

*EEG* electroencephalography, *CT* computer tomography, *CSF* cerebrospinal fluid, *MRI* magnetic resonance imaging, + test or therapy performed

## Discussion

Clinical and radiologic manifestation of PRES is the sole basis for diagnosis due to the absence of uniform diagnostic criteria. The incidence of PRES is still unknown. It has been reported both in children and adults, aged 4 to 90 years [11]. The increased number of new cases of PRES in recent years can be attributed to improved quality and access to diagnostic imaging as well as increasing clinical expertise and awareness [7].

PRES is reported in children less frequently than in adults. It affects children with chronic kidney disease, glomerulonephritis and idiopathic hypertension [12]. Furthermore, PRES was reported in children after erythrocyte transfusion for severe iron deficiency, granulocyte-colony stimulating factor for ulcerative colitis due to neutropenia [12] and peritoneal dialysis [13]. Although all children with malignant diseases are known to be high risk for this pathology [14–17], PRES is most often (55%) described in patients with ALL and significantly less often (9%) in AML [18]. There are also reports of PRES associated with other hemato-oncological diseases, such as Fanconi anaemia, Langerhans histiocytosis, immune haemolytic anaemia [6], Hodgkin lymphoma [14], Non-Hodgkin lymphoma [14] and aplastic anaemia [17]. Steroid treatment or HSCT (haematopoietic stem cell transplantation) constitutes the most common risk factors in this group of patients [16]. According to the literature, PRES is the least likely to occur in children with solid tumours. The prevalence rate in solid tumours is 23% according to de Laat [18] and includes such malignancies as brain tumour [1, 19], osteosarcoma [20], Ewing sarcoma [11, 14, 18], neuroblastoma [20] and hepatoblastoma [21]. Children with solid tumours constituted 50% of our cohort.

The main symptom of PRES in our patients was headache, reported by seven of eight patients. Almost all patients (that is, six) presented with seizures and disorders of consciousness. Mental status alteration secondary to PRES may involve a number of symptoms, such as changes to alertness and activity, lethargy, confusion, somnolence, diminished spontaneity, stupor or even coma. The mental function is slowed. Memory and concentration are impaired [4]. In our cohort, quantitative disorders of consciousness, such as drowsiness and coma, were predominantly seen, but there were three cases of qualitative disorders of consciousness hearing voices and seeing images and flashes of light.

Seizures secondary to PRES may become focal, but are usually generalised, tonic-clonic [22]. Seizures are considered a more common symptom of PRES in children than in adults (94.7 vs 65.9%) [8]. As it is with febrile seizures, also PRES-related seizures are attributable to CNS immaturity and children’s higher susceptibility to blood-brain barrier disruption by toxins or proinflammatory cytokines [10]. According some authors [23, 24], the prevalence of seizures in PRES is higher in younger children as compared to older ones. Our observations do not support this statement; six children in our cohort developed seizures, and the only two who did not were our youngest enrolled patients (a 3-year-old boy with ALL and a 3.5-year-old boy with neuroblastoma).

Patients with PRES often report visual disturbance: hemianopia, diplopia and frank cortical blindness. Some cortically blind patients do not realise that they cannot see (Anton’s syndrome) [4]. In our paper, some degree of visual disturbance was present in four of eight children, only one girl (one of eight) presented with cortical blindness. Visual disturbance is seen less frequently in children with PRES than in adults, potentially due to less frequent involvement of optic radiations and occipital visual cortex in paediatric PRES [8].

In three of our PRES cases, vomiting was present as a sign of elevated intracranial pressure. No focal damage to the central nervous system was diagnosed, both in our study and in others [4, 17].

Clinical manifestation of our patients was generally compatible with the one reported in the literature. According to de Laat et al. [18], who characterised PRES based on 56 childhood cancer patients (seven own patients plus identified published reports), the typical PRES symptoms include seizures (50 children, 89%), altered mental status (20 children, 36%) and headaches (17 children, 30%). In the same research, visual disturbance was observed in 24 (43%) children and cortical blindness in seven (13%) children.

In a majority of published research, PRES occurred in patients with known malignancy during treatment and was therefore considered to be a complication during treatment of childhood cancer [6, 11, 18]. In our research, the time from treatment commencement to the onset of PRES ranged between 6 and 556 days. One of our patients developed seizures secondary to PRES as the first symptom of malignancy. Only one such case of PRES preceding chemotherapy has been reported so far [25]. Clinical symptoms of PRES resolved completely within few days to 4 weeks in most patients.

MRI findings in patients with PRES are not sufficiently specific. They can mimic various neurological conditions such as CSN metastatic disease, cerebral infections, primary CSN tumours and acute drug-induced encephalopathy (after methotrexate, vincristine, ifosfamide). The most characteristic imaging pattern of PRES is associated with the high-signal intensity on T2-weighted images +/or FLAIR MRI of bilateral oedema involving subcortical white matter of the posterior parts of the cerebral hemispheres [4–6].

Computed tomography (CT) findings are often normal or non-specific and may not be sufficient to make the diagnosis [7].

In our sample, all patients (100%) had MRI abnormalities typically located in the parietal and occipital regions. Along with that, against the original description of PRES as posterior encephalopathy syndrome, the atypical MRI presentation included lesions in other cerebral regions such as the cerebellum (two cases), brainstem (one case) and frontal lobes (seven cases). Similar data is provided by other authors—atypical lesions within the frontal lobe are confirmed in 47 [4] to 89% [26] of adults and 54 [27] to 81% [16] of children with PRES. The cerebellar and brainstem involvement were reported in 13–58% of adults [2, 4] and 58% of children [28]. Furthermore, “atypical” cases with isolated cerebellar lesions have been reported in literature [29]. According to some authors, cerebellar involvement is more common in children than in adults. This finding supports the hypothesis of more vulnerable cerebellar circulation in children, due to the lack of posterior sympathetic innervation [30, 31].

PRES-induced abnormalities do not have to be restricted to the white matter. Grey matter involvement was confirmed in five of our patients, who developed seizures. According to the literature, grey matter (cortical) involvement is present in all children [1, 14], but not in all adults (27–44%) with PRES [2, 4, 26], which explains why seizures are more commonly seen in children.

In all cases, the MRI abnormalities were symmetrical and relatively bilateral, as reported by other authors [5, 6, 15, 19]. However, there are reported cases of completely unilateral involvement [2, 30], which pose a diagnostic challenge being difficult to distinguish from infarction.

It should be noted that whilst clinical symptoms resolve quickly and the patient’s condition improves accordingly within hours to days, radiologic abnormalities may persist significantly longer. It is difficult to determine the moment of resolution of radiologic lesions due to the absence of a uniform diagnostic algorithm applicable to PRES. In our paper, follow-up imaging performed in six patients confirmed reversibility of the syndrome in five cases. One remaining patient presented with marked regression of previously reported lesions. The literature search yields highly variable time intervals between diagnostic and follow-up MRI [15, 19, 20, 32]. Only progressive long-term follow-up of children can explain whether PRES is fully reversible.

Hypertension is the key risk factor of PRES, more often seen in children (100% according to Morris [14], 68% according to Kim [16]) than in adults (67% according to Bartynski [10], 84% according to Siebert [8]). Six of our patients presented with hypertension at the onset of PRES, the blood pressure values were very high (> 99 percentile). A boy with ALL developed seizures and status epilepticus as a result of abrupt hypertension; he was treated with steroids, which is a known risk factor for hypertension.

Hypertension in patients with PRES is usually secondary to treatment (steroids, cyclosporin), low serum magnesium levels or renin-angiotesin-aldosteron system response to tumour-compressing renal vasculature. Such renovascular hypertension occurred in two of our patients.

Low serum magnesium level has been reported as a cause of hypertension in two PRES patients. In our cohort, the mean magnesium level was 0.69 mmol/L. The lowest (0.38 mmol/L) serum magnesium level was found in a girl with hepatoblastoma treated with cisplatin, which is a well-known factor causing the urinary excretion of magnesium. The above is in line with the reports by Khan [1] and Bartynsky [10].

However, not all patients with PRES have hypertension. In patients, with PRES and normal blood pressure, cytotoxicity is considered as the reason of the brain oedema [10].

Other reported causes of non-hypertensive PRES include infection, sepsis, septic shock, blood transfusion or administration of granulocyte-colony stimulating factors (GCSF) [10, 16]. Many drugs used during childhood cancer chemotherapy have been linked to PRES, including vincristine [33], cisplatin [34], methotrexate [35], l-asparaginase [26], carboplatin [36], cytarabine [37] and immunosuppressive agents, such as cyclosporin A [4], or tacrolimus [4, 10]. A few cases of PRES related to haematopoietic stem cell transplantation in children have been reported. No single chemotherapeutic agent has been identified as clearly responsible for PRES. The role of steroids is unclear. They may trigger PRES directly or indirectly by contributing to steroid-induced hypertension [19].

There have been no clear uniform treatment guidelines for PRES. Treatment is usually symptomatic. Anticonvulsant treatment duration is debated. Such treatment is discontinued in patients as soon as PRES symptoms have resolved. According to other authors, it should be continued for up to 3 months following symptom resolution [19]. Lucchini et al. [6] suggest anticonvulsant treatment for up to 2 years following PRES resolution in order to prevent potential late complications [6, 18]. Our patients were treated symptomatically with anticonvulsants, diuretics and hypotensives plus—as a part of prevention—also antifungals, antibiotics and antivirals, no longer than 2 months.

No patient had recurrence of PRES, despite continuation of cancer treatment. However, one patient developed refractory epilepsy 5 years after PRES. Two patients died during follow-up. One death was due to *Clostridium* sepsis, and the other one—due to the progression of basal disease—hepatoblastoma. PRES is a risk factor for patient outcome because of the need to suspend chemotherapy when it occurs in induction and obviously because of its proper mortality risk [31]*.* The onset of PRES in our cohort disrupted chemotherapy course postponing it by 0–30 days (mean of 13 days). One patient died due to septicaemia 4 days following onset of PRES, whilst another one was diagnosed with PRES before chemotherapy commencement.

## Conclusions

Although the pathomechanism of PRES has not been fully understood yet, there is an established association between the risk of PRES and a number of different conditions and therapies. As a result, PRES should be included in differential diagnosis in all patients with acute neurological symptoms. Arterial hypertension is a significant risk factor for PRES, hence the need to monitor it in children receiving cancer treatment. Due to PRES symptoms being relatively non-specific, the final diagnosis should be made based on diagnostic imaging.
